# Exceptional Response to Pembrolizumab Following Durvalumab Failure in a Patient With Microsatellite Instability-High Cholangiocarcinoma

**DOI:** 10.7759/cureus.75724

**Published:** 2024-12-15

**Authors:** Akinori Sasaki, Kayo Matsuda, Risa Okamoto

**Affiliations:** 1 Gastroenterology and Oncology, Tokyo Bay Urayasu Ichikawa Medical Center, Urayasu, JPN; 2 Gastroenterology, Tokyo Bay Urayasu Ichikawa Medical Center, Urayasu, JPN

**Keywords:** cholangiocarcinoma, durvalumab, immune checkpoint inhibitor, microsatellite instability-high, pembrolizumab

## Abstract

Typically, patients with advanced cholangiocarcinoma have a poor prognosis because of the limited effective chemotherapy options available. Studies on genotype-directed therapies for cholangiocarcinoma are increasing. However, limited clinical data are currently available for evaluating the efficacy of molecular-targeted therapies. Herein, we report the case of an 83-year-old man diagnosed with microsatellite instability-high (MSI-H) cholangiocarcinoma. MSI-H was detected using comprehensive genomic profiling after resistance to chemotherapy with durvalumab, an anti-programmed death ligand-1 (anti-PD-L1) antibody. Subsequently, the patient received pembrolizumab, a humanized IgG4 monoclonal antibody that targets anti-programmed cell death protein-1 (anti-PD-1). A CT scan after four cycles of pembrolizumab revealed marked shrinkage of the primary tumor. He experienced grade 2 cutaneous immune-related adverse events, but these events improved with topical steroid treatment. The patient is currently being treated with pembrolizumab for at least 10 months and has not exhibited any tumor (A1) progression. To the best of our knowledge, this is the first report on the efficacy of treatment with an anti-PD-1 antibody in a patient with MSI-H cholangiocarcinoma after the failure of the anti-PD-L1 antibody treatment.

## Introduction

Cholangiocarcinoma is a malignant tumor that can be classified into various subtypes based on the site of origin: intrahepatic cholangiocarcinomas, extrahepatic cholangiocarcinomas, gallbladder carcinomas, and ampullary carcinomas [1]. Due to the rapid growth of cholangiocarcinomas, they frequently metastasize to the liver and abdominal lymph nodes and invade major intrahepatic vessels by the time of diagnosis. Therefore, approximately 80% of cholangiocarcinomas are considered inoperable [2]. In cases of advanced cholangiocarcinoma, the current standard of care is systemic chemotherapy. The combination of durvalumab, an immune checkpoint inhibitor (ICI), with conventional gemcitabine and cisplatin therapies has recently been demonstrated to improve overall survival (OS), and the drug has been granted approval for use in treating cholangiocarcinoma [3]. Nevertheless, the prognosis for patients with advanced cholangiocarcinomas remains poor, as the maximum survival rate achieved with the current chemotherapy regimens is 12 to 14 months [3,4].

Recently, genetic profiling has been widely used in patients with malignant tumors, including cholangiocarcinomas. Genetic profiling of patients with cholangiocarcinomas can reveal targetable mutations, such as those in the FGFR2 and IDH1 genes [5,6]. Moreover, several chemotherapies, such as ICIs targeting tumor mutational burden-high (TMB-H) and microsatellite instability-high (MSI-H), can be used regardless of the type of cancer [7,8]. Durvalumab has been approved as first-line therapy for cholangiocarcinoma, and pembrolizumab can be used as second-line therapy in patients with MSI-H or TMB-H cholangiocarcinoma; however, whether pembrolizumab is effective for these patients after durvalumab failure remains unknown. Here, we report a case of MSI-H cholangiocarcinoma in which an exceptional response to pembrolizumab was observed after progression occurred following durvalumab treatment. The patient provided informed consent to present their anonymized clinical information in this report.

## Case presentation

**Figure 1:**
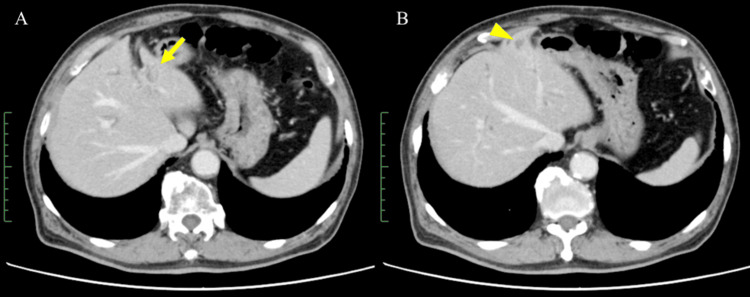
Abdominal computed tomography (CT) images at diagnosis (A, B) Abdominal CT images revealed a mass lesion in the left lobe of the liver (arrow) and dilation of the left intrahepatic bile duct (arrowhead).

**Figure 2:**
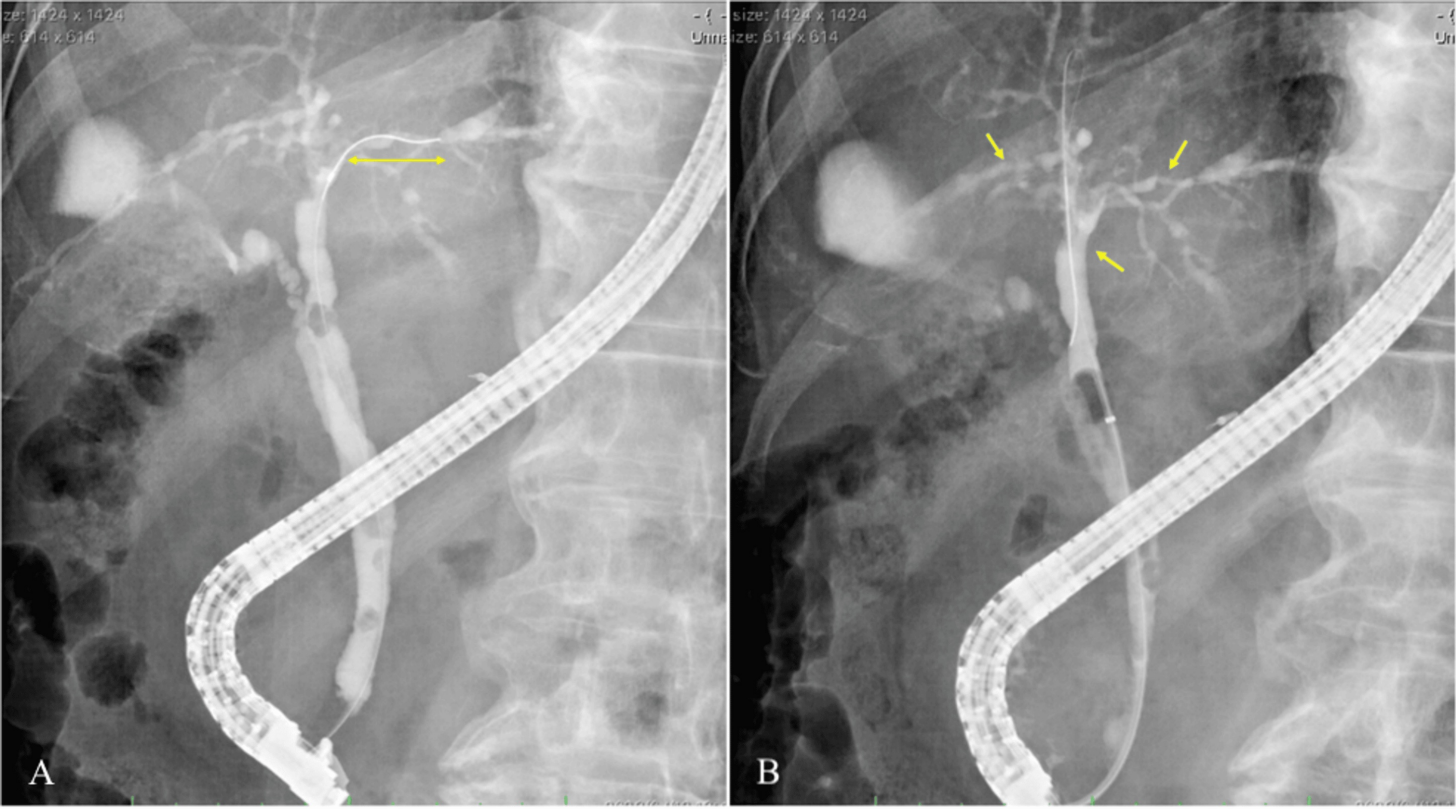
Endoscopic retrograde cholangiopancreatography images at diagnosis (A) Endoscopic retrograde cholangiopancreatography images showed an approximately 4-mm stricture in the left intrahepatic bile duct (two-way arrow), with dilation observed in the peripheral bile ducts. (B) Bile duct biopsies were performed at the sites of stenosis in the right intrahepatic bile duct, common bile duct, and left intraductal bile duct (arrow). Adenocarcinoma was detected at all the sites.

An 83-year-old male was referred to our hospital with complaints of fatigue and loss of appetite, which had persisted for several weeks. The medical history of the patient included dyslipidemia and diabetes. Laboratory tests revealed elevated levels of liver enzymes (aspartate aminotransferase (AST): 39 U/L, alanine transaminase (ALT): 42 U/L, and gamma-glutamyl transpeptidase (γ-GTP): 303 U/L). A mass lesion with a diameter of 1.5 cm in the left lobe of the liver and dilatation of the left intrahepatic bile duct was detected using abdominal computed tomography (CT) (Figure 1). Subsequently, endoscopic retrograde cholangiopancreatography (ERCP) was performed for diagnostic purposes. This revealed an approximately 4-mm stricture in the left intrahepatic bile duct, with dilation observed in the peripheral bile ducts (Figure 2). To evaluate the extent of the lesion, biopsy samples were obtained from the right intrahepatic bile duct and common bile duct under endoscopic guidance. Finally, a biopsy of the left intrahepatic bile duct stricture was performed, and the procedure was concluded. Pathological examination of all the biopsy samples revealed adenocarcinoma, indicating extensive tumor spread. After consultation with the surgical team, it was determined that surgical resection with curative intent was not feasible, and a decision to proceed with chemotherapy was made.

**Figure 3:**
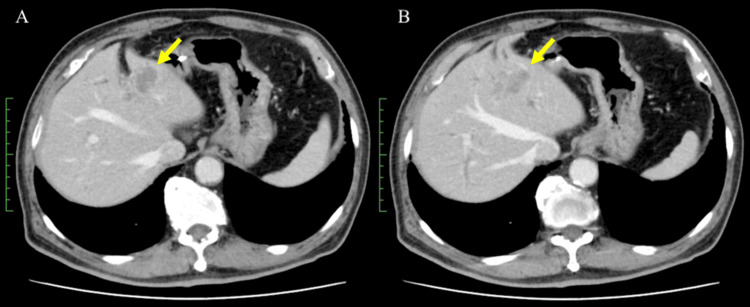
Abdominal computed tomography (CT) images obtained after three cycles of treatment with cisplatin, gemcitabine, and durvalumab (A, B) Abdominal CT images revealed an enlarged primary tumor in the liver (arrow).

The tumor of the patient was deemed inoperable, and he was administered first-line chemotherapy with reduced doses of cisplatin (25 mg/m^2^, days 1 and 8), gemcitabine (800 mg/m^2^, days 1 and 8), and durvalumab (1500 mg/body, day 1) owing to his advanced age. Tumor marker analysis and contrast-enhanced CT were performed after three cycles of first-line chemotherapy. Levels of the tumor marker carbohydrate antigen (CA) 19-9 increased from 130.1 U/mL to 954.8 U/mL, and an abdominal CT scan revealed an enlarged primary tumor in the liver (Figure 3). Based on these results, the patient's condition was considered refractory to treatment with gemcitabine, cisplatin, and durvalumab. Therefore, multigene panel testing was carried out on the tumor tissues from the cholangiocarcinoma by Foundation One CDx (Foundation Medicine, Cambridge, MA, USA). MSI-H (MSH6) is an actionable genomic mutation. Additionally, alterations were detected in the AMER1, ARID1A, and ATM genes. The patient was recommended for pembrolizumab therapy for MSI-H solid tumors based on the results of the multigene panel-based comprehensive genomic profiling (CGP). The patient had already received durvalumab, which was determined to be ineffective. However, because no other treatment options were available, he was started on the ICI pembrolizumab.

**Figure 4:**
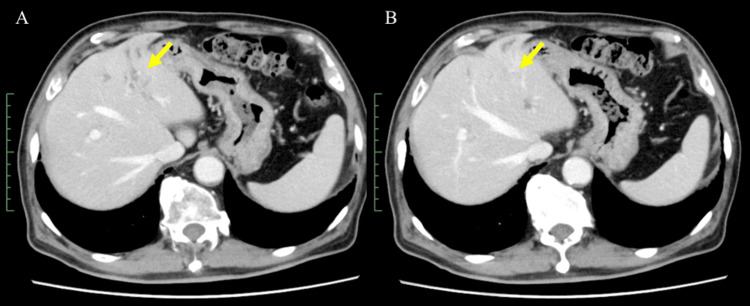
Abdominal computed tomography (CT) images obtained after treatment with pembrolizumab (A, B) Abdominal CT images showed significant shrinkage of the primary lesion (arrow).

Four weeks after the last administration of durvalumab, the patient was treated with pembrolizumab (200 mg every three weeks) as the second-line chemotherapy. He received four cycles of pembrolizumab, and the levels of serum carcinoembryonic antigen and CA 19-9 decreased to normal values (from 954.8 U/mL to 18.5 U/mL) after treatment. A CT scan obtained three months after the treatment showed significant shrinkage of the primary lesion (Figure 4). The patient developed grade 2 cutaneous immune-related adverse events after seven courses of pembrolizumab. His dermatitis improved with topical steroid treatment, and he was able to continue receiving pembrolizumab. To date, the patient has been receiving pembrolizumab for more than 10 months without any tumor progression.

## Discussion

Here, we reported the case of a patient with MSI-H cholangiocarcinoma treated with pembrolizumab after durvalumab treatment failed. MSI-H solid tumors have been shown to respond to ICIs; however, whether the therapeutic effects differ between anti-programmed cell death protein-1 (anti-PD-1) and anti-programmed death ligand-1 (anti-PD-L1) antibodies remains unknown. To the best of our knowledge, this is the first report of an exceptional response to pembrolizumab after the failure of durvalumab in a patient with MSI-H cholangiocarcinoma.

Based on the results of the TOPAZ-1 and KEYNOTE-966 trials, durvalumab and pembrolizumab, both ICIs, are currently available as first-line therapies for biliary tract cancer [3,9]. No direct comparisons have been made regarding the efficacy of these agents. However, the results of each trial showed that the response rates, progression-free survival, and OS were comparable. Patients with MSI-H were included in these studies; however, a subgroup analysis of these patients was not performed because of the small number of patients included in both studies. A previous trial evaluated the treatment response of patients with non-small cell lung cancer to anti-PD-1 antibodies (nivolumab and pembrolizumab) after the failure of anti-PD-L1 antibodies (atezolizumab and durvalumab) [10]. However, the overall response to the anti-PD-1 antibodies retreatment was poor (overall response rate: 0%; progressive disease: 60%). In the context of lung sarcoma-like carcinoma, only one case of response to pembrolizumab after durvalumab treatment failure has been reported [11]. However, no such reports on biliary tract cancer or MSI-H solid tumors have been published.

The mechanisms underlying why patients do not respond to durvalumab but respond to pembrolizumab remain unknown. This may be because durvalumab is an anti-PD-L1 antibody, and pembrolizumab is an anti-PD-1 antibody. Anti-PD-L1 antibodies such as durvalumab promote T-cell activation by binding to PD-L1. In contrast, anti-PD-1 antibodies bind to PD-1 and inhibit the binding of PD-L2 and PD-L1. As a result, T-cell activation may be higher under treatment with anti-PD-1 antibodies than under anti-PD-L1 antibodies. A meta-analysis comparing the efficacy of anti-PD-1 and anti-PD-L1 antibodies reported that anti-PD-1 antibodies resulted in a significantly higher OS than anti-PD-L1 antibodies [12,13]. Furthermore, although not a direct comparison, a study on ICIs in the treatment of MSI-H solid tumors revealed that the response rate of pembrolizumab tended to be higher than that of durvalumab [8,14]. PD-L2 expression is associated with the response to ICIs. A previous study evaluated PD-L2 expression in patients with renal cell carcinoma, melanoma, urothelial carcinoma, and non-small cell lung cancer [15]. In this study, PD-L2 expression was correlated with the efficacy of atezolizumab, an anti-PD-L1 antibody. In addition, a study on head and neck cancer found an association between PD-L2 expression and clinical response to pembrolizumab [16]. The administration of an anti-PD-L1 antibody may not have sufficiently inhibited T-cell activation in our case. However, the anti-tumor effect may have also occurred through PD-1/PD-L2 interaction blockade by pembrolizumab. Dermatitis, an immune-related adverse event, was observed after switching to the anti-PD-1 antibody, suggesting that T-cell activation may have been higher under the anti-PD-1 antibody.

In this case study, we had to decide whether pembrolizumab or S-1 would be optimal after durvalumab was deemed ineffective. Nevertheless, based on the abovementioned information, we hypothesized that pembrolizumab might be effective even after progression occurred with durvalumab. Consequently, the patient was treated with pembrolizumab and achieved a remarkable response to the treatment.

## Conclusions

Our patient showed an exceptional response to pembrolizumab after durvalumab treatment failure. We found that switching from the anti-PD-L1 antibody to anti-PD-1 antibody had an anti-tumor effect. This result suggests that if anti-PD-L1 antibodies are ineffective, switching to anti-PD-1 antibodies may still be effective. Although this is a single case report, it may inform future clinical practice decisions and encourage further clinical trials based on our results.
